# Feature-Based Change Detection Reveals Inconsistent Individual Differences in Visual Working Memory Capacity

**DOI:** 10.3389/fnsys.2016.00033

**Published:** 2016-04-19

**Authors:** Joseph P. Ambrose, Sobanawartiny Wijeakumar, Aaron T. Buss, John P. Spencer

**Affiliations:** ^1^Department of Applied Mathematics and Computational Sciences, University of Iowa, Iowa CityIA, USA; ^2^School of Psychology, University of East AngliaNorwich, UK; ^3^Department of Psychology, University of Tennessee, KnoxvilleTN, USA

**Keywords:** change detection, fMRI, individual differences, visual working memory, working memory capacity

## Abstract

Visual working memory (VWM) is a key cognitive system that enables people to hold visual information in mind after a stimulus has been removed and compare past and present to detect changes that have occurred. VWM is severely capacity limited to around 3–4 items, although there are robust individual differences in this limit. Importantly, these individual differences are evident in neural measures of VWM capacity. Here, we capitalized on recent work showing that capacity is lower for more complex stimulus dimension. In particular, we asked whether individual differences in capacity remain consistent if capacity is shifted by a more demanding task, and, further, whether the correspondence between behavioral and neural measures holds across a shift in VWM capacity. Participants completed a change detection (CD) task with simple colors and complex shapes in an fMRI experiment. As expected, capacity was significantly lower for the shape dimension. Moreover, there were robust individual differences in behavioral estimates of VWM capacity across dimensions. Similarly, participants with a stronger BOLD response for color also showed a strong neural response for shape within the lateral occipital cortex, intraparietal sulcus (IPS), and superior IPS. Although there were robust individual differences in the behavioral and neural measures, we found little evidence of systematic brain-behavior correlations across feature dimensions. This suggests that behavioral and neural measures of capacity provide different views onto the processes that underlie VWM and CD. Recent theoretical approaches that attempt to bridge between behavioral and neural measures are well positioned to address these findings in future work.

## Introduction

Visual working memory (VWM) is a core cognitive system with a highly limited capacity of 3–4 items (Luck and Vogel, 1997). VWM plays a key role in much of visual cognition, comparing percepts that cannot be simultaneously foveated and identifying changes in the world when they occur (Vogel et al., 2001). VWM capacity limitations are reliably associated with individual differences in a host of cognitive functions (Conway et al., 2003), and VWM deficits have been observed in clinical populations, including children diagnosed with autism (Steele et al., 2007) as well as children born preterm (Vicari et al., 2004). VWM appears to be particularly predictive of individual differences in cognitive performance. By some estimates, individual differences in VWM capacity account for up to 40% of the variance in global fluid intelligence (Fukuda et al., 2010).

What neural mechanisms underlie VWM? Research has shown that a distributed network of frontal and posterior cortical regions underlies performance in VWM tasks. In particular, VWM representations are actively maintained in the intraparietal sulcus (IPS), the DLPFC, the ventral-occipital cortex (VOC) for color stimuli, and the lateral-occipital complex (LOC) for shape stimuli (Todd and Marois, 2004, 2005). In addition, there is suppression of the temporo-parietal junction (TPJ) during the delay interval, and activation of the ACC during the comparison phase (Todd et al., 2005; Mitchell and Cusack, 2008).

One of the more striking findings in the fMRI literature is that the BOLD response increases as the memory load is varied from 1 to 3 items and then asymptotes at higher loads (Harrison et al., 2010). This occurs within critical parts of the VWM network including the IPS and VOC (Todd and Marois, 2004). What is striking about these data is that they correspond with behavioral estimates of VWM capacity: estimates suggest that people can hold approximately 3–4 items in VWM (Luck and Vogel, 1997; Vogel et al., 2001). Thus, there is an apparent correspondence between *neural* capacity as indicated by the asymptotic BOLD pattern and *behavioral* capacity as indicated by measures such as Pashler’s *K* (Pashler, 1988).

Evidence supporting this relationship comes from Todd and Marois (2005). They found a significant correlation between behavioral estimates of capacity and a normalized BOLD signal in posterior parietal cortex measured at the set size associated with each participant’s capacity. There was also a significant correlation between behavioral capacity and neural capacity in VOC during the maintenance phase of one experiment. These data are consistent with ERP data from Vogel and Machizawa (2004) showing similar correlations over parietal and occipital cortex. Interestingly, correlations with behavioral capacity estimates were not pervasive: no significant correlations with behavior were observed in anterior cingulate cortex or in middle frontal gyrus.

Given the specificity of these findings to two neural loci, we sought to examine the robustness of the relationship between behavioral estimates of capacity and neural estimates of capacity, taking advantage of recent findings. In particular, Song and Jiang (2006) examined the neural bases of VWM by examining performance in a change detection (CD) task as people remembered colors, shapes, or both feature dimensions. Consistent with Alvarez and Cavanagh (2004), they found capacity differences for colors and shapes: participants remembered 3–4 colors but only 1–2 shapes. They also found the neural asymptotic pattern for both color and shape stimuli across multiple sites within the VWM network, with a stronger BOLD response for shapes than for colors.

These data set the stage for the individual differences approach in the present study. In particular, we asked whether the correlation between behavioral capacity and neural capacity for simple colors also holds for shapes despite dramatic differences in capacity for the two stimulus dimensions. That is, will individuals with a high capacity for colors also have high capacity for shapes and, critically, will correlations between behavioral and neural capacity measures hold despite dramatic differences in capacity across dimensions? Such a result would suggest a very strong link between behavioral capacity and neural capacity.

To test this question, we used a within-subjects design. Participants completed a VWM task with simple colors on one fMRI scanning day, and a VWM task with shapes on a second scanning day. We chose to use shapes from Drucker and Aguirre’s (2009) study on shape similarity because these shapes have good psychometric properties (Zahn and Roskies, 1972) and have been well localized with fMRI. We estimated participants’ VWM capacity along each dimension from their behavioral performance and examined whether behavioral estimates of capacity across dimensions were robust within individuals. Similarly, we measured neural capacity for each dimension across 30 ROIs identified from a recent meta-analysis of the VWM fMRI literature (Wijeakumar et al., 2015) as well as from Drucker and Aguirre (2009), and examined whether neural estimates of capacity across dimensions were robust within individuals. Finally, we examined correlations between the behavioral and neural capacity measures to determine whether there were robust individual differences between brain and behavior and whether these relationships remained robust across dimensions despite large differences in VWM capacity.

## Materials and Methods

### Participants

Twenty right-handed native English-speaking subjects took part in the experiment (age range 25 ± 4 years; 11 men, 9 women). All participants were recruited from the University of Iowa campus and community. All participants had normal or corrected-to-normal vision. All participants signed an informed consent document approved by the Ethics Committee at the University of Iowa.

We acknowledge that the low sample size is a limitation of this study. However, we note that this limitation is common in fMRI studies due to resource limitations. For example, the motivating studies by Alvarez and Cavanagh (2004) and Song and Jiang (2006) had a sample size of 12 and 6, respectively.

### Procedure

The experimental paradigms were created using E-prime version 2.0 and were run on an HP computer (Windows 7). We used two variants of a CD task. In the Color CD task, the shapes of the stimuli were held constant. Participants were shown a memory array of 1–6 colored stimuli (Set Size). After a brief delay, they were shown a test array that was either the same array (Same condition) or an array where one of the stimuli had a different color (Different condition). In the Shape CD task, the colors of the stimuli were held constant. Participants were shown a memory array of 1–6 stimuli. After a delay, they were shown either the same array (Same condition) or an array where one of the stimuli had a different shape (Different condition). Participants were asked to indicate if the items were the same or different using the index or middle finger buttons on a right-handed manipulandam box. At the start of the task, they were informed which button to push to indicate a Same response versus a Different response. There were no practice trials, but participants were shown example sequences during screening to familiarize them with the task before entering the scanner.

Colors were equally distributed in CIELAB 1976 color space. Shapes were based on Drucker and Aguirre’s (2009) RFC-defined stimuli. Sets of eight possible colors and shapes used in the task were generated so that each color and shape were separated by 45° in feature space. Items were randomly selected from this pool to construct the stimulus array on each trial. The changed feature was also drawn from this pool during Different trials.

Each trial began with the presentation of a fixation cross for 2500 ms, followed by the memory array for 500 ms, then a blank screen delay for 1200 ms, and finally the test array for 1500 ms. The inter-trial interval was jittered between 1000 ms (50% of trials), 2500 ms (25%), and 3500 ms (25%). Participants were instructed to respond as quickly and accurately as possible. If a response was not entered within the duration of the test array’s presentation, ‘No Response Detected’ was displayed on the screen, and the trial was excluded from analysis.

### Design

Participants completed a total of four runs each for the Color and Shape CD tasks. Each set of runs occurred over a single scanning block with separate dimensions on separate days. The order of the scanning days (Color first versus Shape first) was counterbalanced across participants. Each run consisted of 20 randomized trials (10 Same, 10 Different) at each set size (SS1–6) completed in increasing order. The goal of increasing set size across blocks was to maximize stability in the measurements of performance at each set size. Moreover, we hoped that the systematic ordering would help participants remain engaged throughout the experiment.

### Image Acquisition and Processing

A 3T Siemens TIM Trio magnetic resonance imaging system with a 12-channel head coil located at the University of Iowa’s Magnetic Resonance Research Facility was used. Anatomical T1 weighted volumes were collected using an MP-RAGE sequence. Functional BOLD imaging was acquired using an axial 2D echo-planar gradient echo sequence with the following parameters: TE = 30 ms, TR = 2000 ms, flip angle = 70°, FOV = 240 mm × 240 mm, matrix = 64 × 64, slice thickness/gap = 4.0/1.0 mm, and bandwidth = 1920 Hz/pixel. Each run was approximately 16 min and collected 491 volumes.

Head movement during the experiment was restricted using foam padding inserted between the observer’s head and the head coil. The tasks were presented using E-prime software and a high-resolution projection system. The stimuli were subtended at a visual angle of 3.2–4.2°. In each trial, the stimuli were randomly arranged between six equidistant positions centered on a virtual circle with a visual angle of 6.7° from the center of the screen. Responses were recorded by a manipulandum strapped to the participants’ hands. The timing of the presented stimuli was synchronized to the trigger pulse from the MRI scanner. Data were analyzed using Analysis of Functional NeuroImages (AFNIs) software. Standard preprocessing was used that included slice timing correction, outlier removal, motion correction, and spatial smoothing (Gaussian FWHM = 8 mm).

### Methods of Analysis

Behavioral performance was assessed using Pashler’s K which provides a behavioral index of VWM capacity at each set size (Pashler, 1988). Formally, this is given by the formula $k=N\,\frac{\left(h-f\right)}{\left(1-f\right)}$ where *N* is the set size, *h* is the hit rate (rate of correct different trials), and *f* is the false alarm rate (rate of incorrect same trials). Note that Pashler’s K is the measure of choice when using a whole array test. Each participant was assigned a capacity value for each dimension by selecting the maximum *K* value across set sizes for that dimension. Given that point estimates can provide a noisy estimate of performance when values are quite comparable (as we expected would be the case at high set sizes), we also fit the K function with linear and quadratic functions for each dimension and selected the functional form that fit the data best. We then used the coefficient estimates from the fit as a secondary behavioral measure.

ROI-based analyses were carried out using 10 mm spherical regions defined using coordinates from regions of interest from the VWM literature (see e.g., Pessoa et al., 2002; Todd and Marois, 2004; Harrison et al., 2010). In particular, we focused on 21 ROIs from a recent meta-analysis (Wijeakumar et al., 2015); nine more were added from Drucker and Aguirre (2009) to examine cortical regions that might be selective for processing stimulus shape. Average beta values were extracted for each ROI (1–30), set size (1–6), and feature (Color, Shape) for each participant. Only trials with correct responses were included in the analyses as the number of incorrect trials for some of the lower set sizes was too small to analyze.

A 2-factor (set size, feature) ANOVA was carried out on data from each ROI to identify ROIs that showed a change in the BOLD response across set sizes. We then conducted additional analyses on the set of ROIs with Set Size or Set Size × Feature interactions. In particular, for each included ROI, we computed the maximum BOLD signal across set sizes for each dimension and the BOLD signal at the set size that matched the maximum *K* value for each subject and dimension. Finally, we examined correlations within and between the behavioral and neural measures using Pearson’s correlation to examine whether behavioral estimates of capacity and neural estimates of capacity are correlated within individuals and across dimensions.

## Results

### Behavioral Results

**FIGURE 1:**
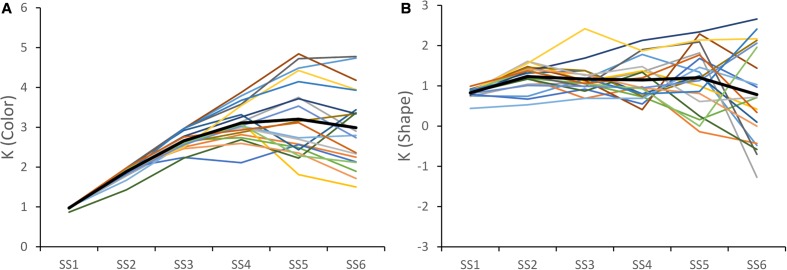
***K* values for each subject across set size for color **(A)** and shape **(B)** trials.** The solid black line shows the average *K* values over subjects.

*K* values were estimated for each set size, participant, and stimulus dimension. **Figure 1** shows these *K* values across participants for the color (left panel) and shape (right panel) dimensions. As is evident, there were differences across stimulus dimensions. In the Color CD task, participants generally had higher *K* values (note that we scaled the panels differently to highlight the individual differences across participants). Indeed, across the sample, the Max *K* value for color was significantly greater than the Max K for Shape, *t*(19) = 13.495, *p* < 0.001. The Color *K* values were also less variable across set sizes showing a clear increasing and then decreasing pattern. By contrast, performance in Shape CD declined less at higher set sizes, reflecting the difficulty participants had with the Shape CD task beyond the lowest set sizes.

**FIGURE 2:**
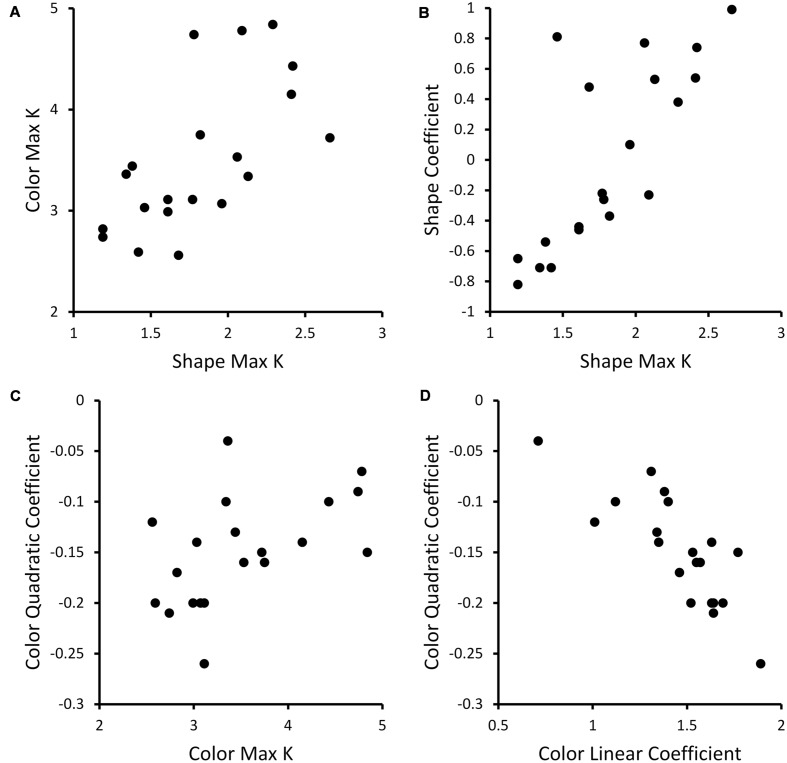
**Scatterplots showing correlations between behavioral measures across participants.** **(A)** Scatterplot showing Color Max *K* and Shape Max *K* values across participants. **(B)** Scatterplot of relationship between Shape Max K and linear coefficient of fits of Shape K functions for each individual across set sizes. **(C)** Scatterplot of relationship between Color Max K and quadratic coefficients of fits of Color K functions for each individual across set size. **(D)** Scatterplot showing linear versus quadratic coefficients for quadratic fits of Color K functions across set size.

The other key result from **Figure 1**: participants showed clear individual differences. To examine whether these individual differences were consistent across dimensions, we correlated the Max *K* values across dimensions. There was a significant correlation, *r* = 0.64, *p* < 0.005, indicating that participants with a high capacity for colors generally also had a high capacity for shapes (see **Figure 2A**).

One limitation of the Max K measure is that it only considers a single value of the *K* function to represent each participant’s performance. As an alternative, we fit the data in **Figure 1** with linear and quadratic functions, obtaining coefficient values describing the linear or quadratic fit for each participant and dimension. For Color, we determined that quadratic functions generally provided a better fit of participants’ data than linear functions (the F-change statistic was significant for the quadratic fit for 13 of 20 participants). For Shape, we found that linear functions provided the most parsimonious description of the K functions (only 1 F-change statistic was significant for the quadratic fit). Based on these results, we carried forward the two coefficients from the quadratic fit of Color K and one coefficient from the linear fit of Shape K for each participant for further analysis.

Given that Max K is the most commonly used measure of capacity in the literature, we correlated the quadratic (Color) and linear (Shape) coefficients with Max K to examine the relationship between these measures. There was a significant positive correlation, *r* = 0.50, *p* < 0.05, between the quadratic coefficient and Max K for Color – participants with a strong negative quadratic coefficient who generally performed poorly at high set sizes had lower Max K, while participants with less negative quadratic coefficients (e.g., near -0.1) had higher Max K (see **Figure 2C**). Note that the linear and quadratic coefficients for Color were negatively correlated, *r* = -0.82, *p* < 0.001 (see **Figure 2D**). This linear term serves to shift the peak of the quadratic function so that the fit does not fall off until the K function does – around Set Size 4. For Shape, there was a significant positive correlation, *r* = 0.76, *p* < 0.001, between the linear coefficient and Max K (see **Figure 2B**). Thus, participants with higher capacity tended to show an increase in performance across set size while lower capacity subjects showed no improvement or a decline across set size.

### fMRI Results

As a preliminary step in the fMRI analysis, we determined which of the 30 ROIs identified from the VWM literature were responsive to the memory load manipulation. To this end, we conducted a two-factor (Set Size, Dimension) ANOVA on data from each ROI. Eight ROIs (five from the meta-analysis, three from Drucker and Aguirre, 2009) showed a significant effect of Set Size or an interaction between Set Size and Dimension – left Temporo-Parietal Junction (LTPJ), left Occipital Cortex (LOCC), left Ventral Occipital Cortex (LVOC), right Intraparietal Sulcus (RIPS), right Superior Intraparietal Sulcus (RsIPS), right face-selective Middle Fusiform Gyrus (RfsMFG), and left and right V3a (LV3a, RV3a). Only average beta values from these eight ROIs were included in further analyses.

**FIGURE 3:**
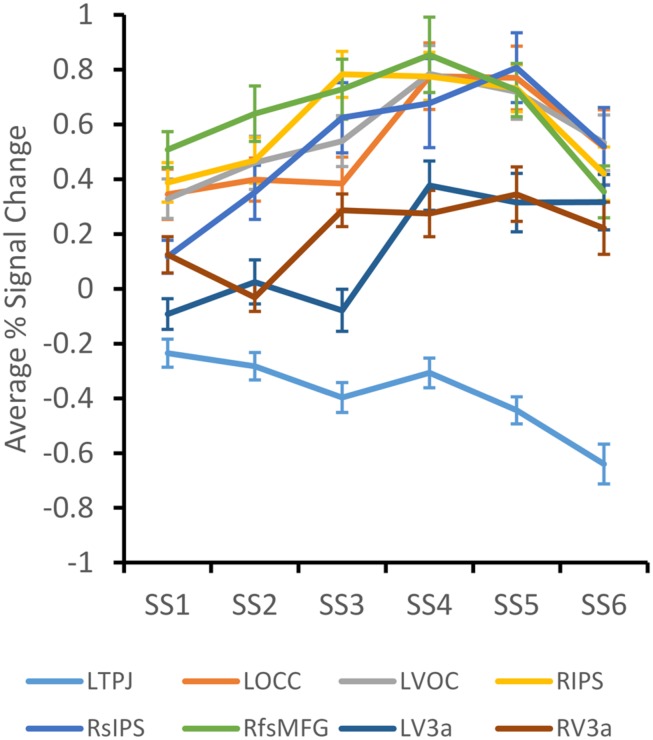
**Average percent BOLD signal change across set size for each ROI that demonstrated a significant effect of Set Size.** Error bars depict ± 1/2 SE.

**Figure 3** shows average percent signal change across the set size manipulation for each cluster. LTPJ was the only cluster to show a decline in the BOLD response across Set Size, *F*(5,95) = 2.71, *p* < 0.05, replicating findings reported by Todd and Marois (2005). Note that there were no significant differences in the LTPJ response across stimulus dimensions. Additionally, V3a showed a very gradual increase in the BOLD response across set size, *F*(5,95) = 2.68, *p* < 0.05. Once again, there were no significant differences in the V3a response across stimulus dimensions, although the BOLD response was generally higher for Shape than for Color [*F*(1,95) = 3.29, *p* = 0.085].

**FIGURE 4:**
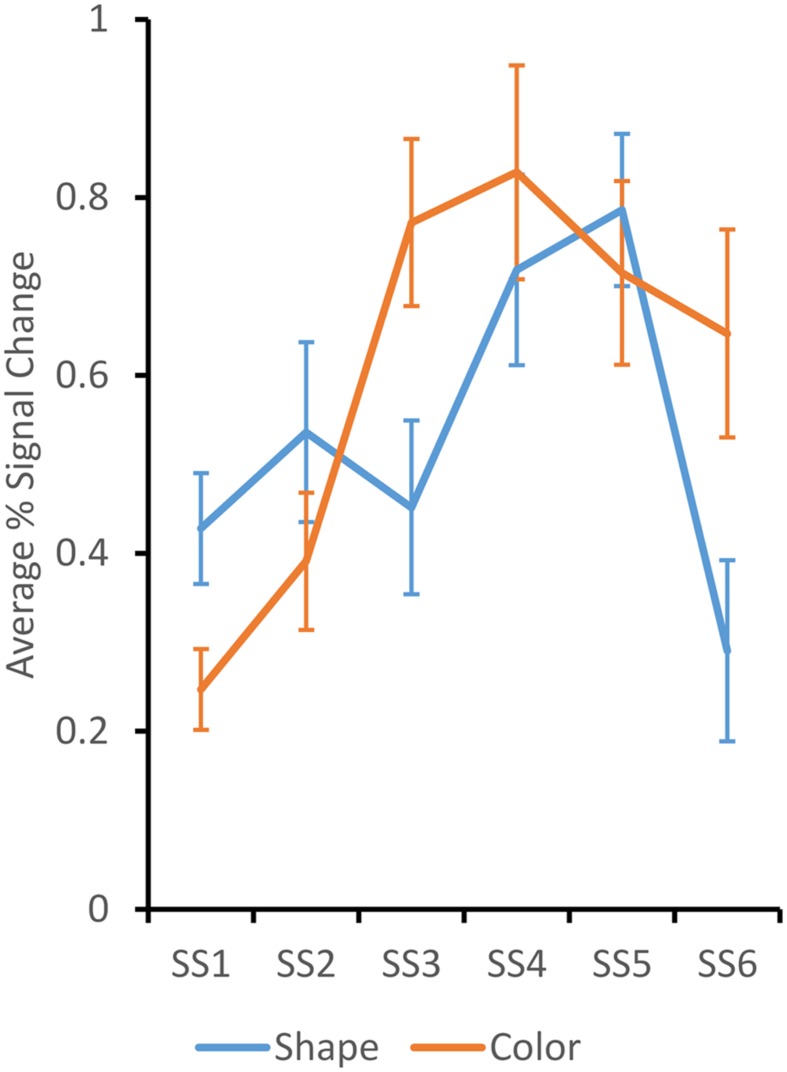
**Average percent BOLD signal change over the LOCC, LVOC, RIPS, RsIPS, and RfsMFG ROIs across set size for shape (blue) and color (orange) trials.** Responses were significantly higher for color at set sizes 3 and 6. Error bars depict ± 1/2 SE.

The remaining five clusters showed an increasing pattern across set size, with a decline at set size 6. Data from these clusters were analyzed together in a three-factor ANOVA with Set Size, Dimension, and Cluster as factors. There was a significant main effect of SS, *F*(5,380) = 4.48, *p* < 0.001, and a significant SS × Dimension interaction, *F*(5,380) = 2.40, *p* < 0.05. The interaction effect is shown in **Figure 4**. The BOLD response for the Color dimension rises more steeply and remains high across set sizes 3–6. By contrast, the BOLD response for the Shape dimension rises more gradually and falls off dramatically at set size 6. *Post hoc* tests determined that the BOLD response for the Color dimension was significantly greater than the Shape dimension at SS3 and SS6, *p* < 0.05. This is consistent with behavioral results that showed greater Max K for Color than for Shape.

In the previous section, we reported that individual differences in Max K for color were correlated with individual differences in Max K for Shape. Do these individual differences hold at the level of the brain as well? To investigate this issue, we measured the maximum BOLD response within each cluster across set sizes for each participant and dimension as well as the BOLD response at the set size at which the maximum *K* value occurred. We then correlated the neural measures. As can be seen in **Table 1**, the Max signal and Max K signal measures are highly correlated within dimensions for 14 of 16 comparisons across clusters. The two comparisons that did not reach significance were both along the shape dimension.

**Table 1:** Correlation scores for neural measures across participants.

		Shape Max	Color Max	Shape at Max K SS	Color at Max K SS
LTPJ	S Max		-0.02	0.66ˆ**	0.21
	C Max			0.20	0.81ˆ**
	S Max K SS				0.18
LOCC	S Max		0.24	0.66ˆ**	0.29
	C Max			0.28	0.96ˆ**
	S Max K SS				0.29
LVOC	S Max		0.70ˆ**	0.36	0.57ˆ**
	C Max			0.34	0.88ˆ**
	S Max K SS				0.09
RIPS	S Max		0.45ˆ*	0.83ˆ**	0.09
	C Max			0.42	0.74ˆ**
	S Max K SS				0.20
RsIPS	S Max		0.40	0.82ˆ**	0.60ˆ**
	C Max			0.36	0.88ˆ**
	S Max K SS				0.55ˆ*
RfsMFG	S Max		0.35	0.72ˆ**	0.37
	C Max			0.18	0.83ˆ**
	S Max K SS				0.08
LV3a	S Max		0.13	0.89ˆ**	0.14
	C Max			0.06	0.72ˆ**
	S Max K SS				0.31
RV3a	S Max		0.04	0.36	0.18
	C Max			0.19	0.55ˆ*
	S Max K SS				0.34


*^∗^Correlation is significant at the 0.05 level. ^∗∗^Correlation is significant at the 0.01 level.*

**FIGURE 5:**
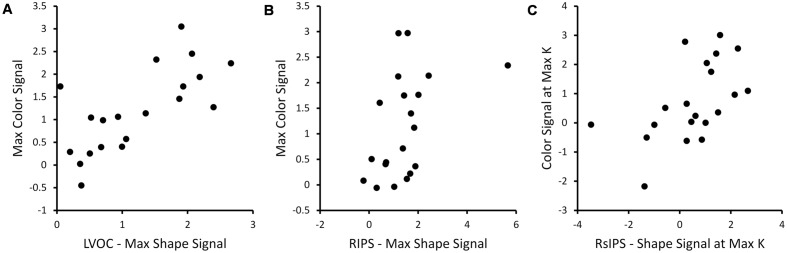
**Exemplar scatterplots showing relationships between neural measures that were significantly correlated across dimensions for the three ROIs with significant correlational patterns (see **Table 1**): **(A)** LVOC, **(B)** RIPS, **(C)** RsIPS**.

The measures were also compared across dimensions. There were significant cross-dimension correlations in VOC, RIPS, and RsIPS (see **Figure 5**). In VOC and RIPS, the Max BOLD responses were correlated across dimensions, while in RsIPS, multiple significant correlations were observed. Thus, in these areas, participants with stronger neural responses when remembering items that varied along one dimension, also tended to have stronger neural responses when remembering stimuli along the other dimension.

### Brain-Behavioral Correlations

The central question in this study was whether individual differences in behavioral capacity were correlated with individual differences in neural capacity and, further, whether these correlations held despite differences in capacity across stimulus dimensions. To examine this question, we correlated the five behavioral measures (Max K for Shape, Max K for Color, the linear coefficient for Shape, and the linear and quadratic coefficients for Color) with the four neural measures (Max BOLD for Shape/Color, BOLD at Max K SS for Shape/Color) within the eight clusters showing statistically robust differences in the neural response across set sizes. **Table 2** shows the results.

**Table 2:** Correlations between behavioral and neural (light gray shading indicates a correlation in a direction opposite of what was expected).

		Shape Max K	Color Max K	Shape coeff	Color linear coeff	Color quadratic coeff
LTPJ	S Max	-0.04	0.08	0.30	-0.30	0.31
	C Max	0.27	0.15	0.15	-0.11	0.19
	S Max K SS	-0.25	-0.33	0.06	-0.20	0.01
	C Max K SS	0.25	0.05	0.33	-0.14	0.14
LOCC	S Max	-0.30	-0.20	-0.38	0.28	-0.38
	C Max	0.21	0.17	0.25	-0.08	0.15
	S Max K SS	-0.28	-0.11	-0.49^∗^	0.12	-0.20
	C Max K SS	0.20	0.13	0.24	0.10	-0.01
LVOC	S Max	-0.43	-0.14	-0.49^∗^	0.07	-0.18
	C Max	-0.03	0.14	-0.19	0.03	0.01
	S Max K SS	-0.37	-0.09	-0.53^∗^	-0.22	0.08
	C Max K SS	0.05	0.07	-0.04	0.12	-0.08
RIPS	S Max	-0.26	-0.10	-0.23	0.07	-0.11
	C Max	-0.02	-0.01	-0.01	0.14	-0.12
	S Max K SS	-0.30	-0.16	-0.31	0.08	-0.15
	C Max K SS	-0.14	-0.27	-0.7	0.21	-0.33
RsIPS	S Max	0.01	-0.02	-0.22	0.39	-0.37
	C Max	0.26	0.11	0.12	0.22	-0.12
	S Max K SS	0.11	0.00	-0.17	0.39	-0.35
	C Max K SS	0.15	-0.11	0.08	0.28	-0.31
RfsMFG	S Max	-0.19	-0.02	-0.28	0.05	-0.06
	C Max	0.23	0.16	0.04	0.19	-0.09
	S Max K SS	-0.23	0.00	-0.40	0.12	-0.10
	C Max K SS	-0.01	-0.12	-0.12	0.17	-0.22
LV3a	S Max	-0.10	0.07	0.11	-0.06	0.08
	C Max	0.00	0.00	-0.01	-0.19	0.18
	S Max K SS	-0.11	-0.02	0.12	0.08	-0.08
	C Max K SS	-0.26	-0.25	-0.19	-0.02	-0.12
RV3a	S Max	-0.17	0.02	-0.04	-0.27	0.22
	C Max	0.37	0.35	0.25	-0.06	0.25
	S Max K SS	-0.17	0.16	-0.48^∗^	-0.39	0.37
	C Max K SS	0.15	0.17	0.15	-0.06	0.11


*^∗^Correlation is significant at the 0.05 level.*

The first striking result is that there were no significant brain-behavior correlations with the Max K measures. The absence of any significant correlations between the standard behavioral capacity measure (K) and neural capacity measures is not consistent with previous findings (Vogel and Machizawa, 2004; Todd and Marois, 2005).

One limitation of Max K is that it is a point estimate of a function. In this context, it is interesting that there were multiple significant correlations between the neural data and coefficients from the curve fits. Nevertheless, brain-behavior correlations for the curve fits for Shape were all in the opposite direction of what was expected (see light gray shading). In particular, the four significant correlations with the linear coefficient for Shape were negative, that is, the stronger the BOLD response for Shape, the shallower the slope of the K function for Shape across set sizes. As with the Max K measure, there were no significant correlations between the behavioral curve fits and the neural measures for Color.

## Discussion

The central goal of this study was to investigate the relationship between behavioral estimates of VWM capacity and neural estimates of VWM capacity using an individual differences approach. In particular, we conducted an fMRI experiment where we varied the complexity of the stimulus dimensions participants had to remember. Based on findings from Song and Jiang (2006), we expected that this would shift VWM capacity between dimensions. The question was whether high capacity individuals for one dimension would remain high capacity individual for the second dimension, and, further, whether brain-behavior correlations would remain robust across this shift in capacity.

Behavioral results from this study were consistent with the expected shift in VWM capacity across dimensions. In particular, capacity for colors was higher and less variable than capacity for shape. In addition, there were robust individual differences in capacity across dimensions: participants with a high capacity for color also had high capacity for shape. Thus, we succeeded in shifting behavioral capacity across dimensions, replicating findings from Song and Jiang (2006; see also, Alvarez and Cavanagh, 2004). We also calculated secondary measures of behavioral capacity by fitting participants’ K functions to linear and quadratic functions – quadratic for Color, linear for Shape. These novel behavioral measures were correlated with Max K. In particular, Max K was positively correlated with the quadratic fit coefficients for Color and linear coefficients for Shape, and negatively correlated with the linear fit coefficients for Color.

We then used an ROI approach to identify brain areas that showed a statistically robust change over set size. ANOVA results replicated several key effects in the VWM and change detection literatures. In particular, we replicated the suppression in LTPJ as the memory load increased (Todd and Marois, 2005). We also found load-dependent responses in RIPS, RsIPS, LOCC, and LVOC (see, e.g., Todd and Marois, 2004; Song and Jiang, 2006; Harrison et al., 2010). Moreover, when the V3a areas were analyzed together, we found a weak dimension effect (*p* = 0.085) with a stronger neural response for Shape versus Color. This is consistent with findings from Drucker and Aguirre (2009). Results from the group analyses also revealed that Color showed a more robust neural response across set sizes than Shape. In particular, BOLD activation rose more quickly over set sizes and reached a more robust asymptote in LOCC, LVOC, RIPS, RsIPS, and RfsMFG. These results are not consistent with Song and Jiang’s (2006) results – they reported greater BOLD activation for shapes than colors in superior parietal lobule, lateral occipital complex, and frontal eye fields. It is possible that this reflects differences in the shapes used across studies. Moreover, Song and Jiang presented variations in color and shape on each trial, asking participants to selectively attend to one dimension or the other. By contrast, we held one dimension constant while varying the other. Although our findings across dimensions clearly differ, there was a consistency across studies: Song and Jiang found a reduction in the BOLD response for shape at high set sizes, similar to the decrease observed at set size 6 here. This reduction in the BOLD response at high set sizes has also been observed with young children (Buss et al., 2014).

To analyze individual differences at the neural level, we extracted Max BOLD and BOLD at Max K SS measures from the ROI data. Within dimension, these measures were highly correlated with each other across all ROIs. Moreover, there were robust individual differences across dimensions in VOC, RIPS, and RsIPS: participants with strong neural responses to Color also had strong neural responses for Shape. Thus, individual differences at the neural level were preserved across dimensions even though there was a significant reduction in capacity moving from Color to Shape. RIPS and RsIPS have been identified in previous studies to represent the spatial positions of objects in VWM (Harrison et al., 2010), possibly binding features together via virtue of their shared spatial positions. If these areas provide a general index of bound object representations, it might explain the robust correlations across dimensions in that high capacity individuals would be expected to have robust object representations regardless of the featural content.

In the final analysis step, we examined whether individual differences in the behavioral measures were related to individual differences in the neural measures. There were no significant correlations with Max K; this was surprising given previous results (Vogel and Machizawa, 2004; Todd and Marois, 2005). Note that Todd and Marois (2005) reported significant correlations between Max K and a normalized BOLD signal in both IPS and VOC. We examined whether normalizing our BOLD data would have an impact; this was not the case. We also examined whether curve fitting of the BOLD data across set size might yield a replication of Todd and Marois’ findings. Once again, this was not the case.

We did find several cases of the opposite correlational pattern where a stronger BOLD response for shape was correlated with an index of *lower* behavioral capacity. It is possible that this reflects selective color processing in two of these areas – LOCC and LVOC. That is, if these areas are selective for color processing, one might expect that greater BOLD activation on shape trials in these areas might be indicative of poorer performance. By contrast, given that RV3a is a shape-selective area, it is not clear why we found a negative correlation between behavioral and neural capacity for shape VWM in this area.

Note that our report is not the first to find a mixed pattern of results when comparing individual differences in VWM capacity across behavioral and neural levels. Todd and Marois (2005) reported robust correlations between behavioral estimates of capacity and IPS activity; however, correlations with VOC activity were only significant in one experiment. Correlations with BOLD responses from all other ROIs were not significant. Critically, both studies had relatively limited sample size for investigations of individual differences (20 in the present report; 17 in Todd and Marois, 2005). This may have contributed to the sparse brain-behavior correlations.

Our conclusion from the present study is that there is a complex relationship between behavioral capacity and neural capacity. This is consistent with recent theoretical work. For instance, Johnson et al. (2014) used a dynamic neural field model of VWM to bridge between the behavioral and neural levels. Their model successfully reproduced patterns of behavioral data across set sizes in detail, including performance on correct and incorrect trials (see also, Johnson et al., 2009a,b). They also found an asymptote in neural activation over set sizes for some neural measures. Nevertheless, there was not a one-to-one relationship between behavioral estimates of capacity and the number of neural representations actively maintained by the model. That is, models with a behavioral capacity of 3–4 items often actively maintained 4–6 items in VWM.

Importantly, recent work has demonstrated that dynamic field models can provide useful insights into individual differences as well (see Perone and Spencer, 2013, 2014). Moreover, we have developed a method to simulate hemodynamics directly from dynamic field models (Buss et al., 2013). These two innovations suggest that dynamic field theory could be a useful theoretical framework to explore the relationship between behavioral and neural VWM capacity in greater detail. This will be a target of future work.

In summary, our results provide evidence that individual differences in both behavioral and neural measures are preserved across shifts in capacity created by processing simple versus complex features. Further, our results provide some evidence that higher capacity individuals determined by behavioral measures are also higher capacity individuals at the neural level. Nevertheless, there is clearly a complex relationship between behavioral estimates of capacity and neural estimates of capacity. Future work will be needed to clarify this relationship, and we suggest that recent neurally grounded theories of VWM might prove useful on this front.

## Author Contributions

JA is the primary author of the manuscript. JS and AB designed the experiment. JA, JS, and SW analyzed and interpreted data from the experiment. JS, SW, and AB all provided important revisions to the manuscript.

## Conflict of Interest Statement

The authors declare that the research was conducted in the absence of any commercial or financial relationships that could be construed as a potential conflict of interest.
